# Synthesis and Evaluation of Pyrimidine Steroids as Antiproliferative Agents

**DOI:** 10.3390/molecules24203676

**Published:** 2019-10-12

**Authors:** Alejandra Cortés-Percino, José Luis Vega-Báez, Anabel Romero-López, Adrián Puerta, Penélope Merino-Montiel, Socorro Meza-Reyes, José M. Padrón, Sara Montiel-Smith

**Affiliations:** 1 Facultad de Ciencias Químicas, Benemérita Universidad Autónoma de Puebla, Ciudad Universitaria, 72570 Puebla, Pue., Mexico; ale891930@hotmail.com (A.C.-P.); jose.vega@correo.buap.mx (J.L.V.-B.); penelope.merino@correo.buap.mx (P.M.-M.); maria.meza@correo.buap.mx (S.M.-R.); 2 Instituto de Física “Luis Rivera Terrazas” Benemérita Universidad Autónoma de Puebla Ecocampus Valsequillo, 72960 San Pedro Zacachimalpa, Pue., Mexico; anabelrl@ifuap.buap.mx; 3 BioLab, Instituto Universitario de Bio-Orgánica “Antonio González” (IUBO-AG), Centro de Investigaciones Biomédicas de Canarias (CIBICAN), Universidad de La Laguna, c/ Astrofísico Francisco Sánchez 2, 38206 La Laguna, Spain; apuertaarocha@gmail.com

**Keywords:** steroids, pyrimidine, heterocycle, cycloaddition reactions, antiproliferative activity

## Abstract

A small and focused library of steroidal non-fused and fused pyrimidines was prepared from pregnenolone acetate and diosgenin, respectively. The key step was the cycloaddition reaction of nitrogen-containing 1,3-binucleophiles with the steroidal α,β-unsaturated ketone. Urea, thiourea and guanidine reacted in a similar manner and afforded the steroidal pyrimidines in good yields. The antiproliferative tests against human tumor cell lines gave GI_50_ values in the micromolar range and had no effect on healthy fibroblasts. Additional experiments indicated that the compounds did not act as P-glycoprotein substrates, thus avoiding the rise of drug resistance. The fused steroidal pyrimidinethione was selected as drug lead for further testing due to its strong antiproliferative activities within the low micromolar range.

## 1. Introduction

The direct use of natural products as drugs is limited because their physicochemical properties are often far from ideal. However, they represent an excellent starting point in drug discovery programs. In fact, 50% of approved drugs have a natural origin and many synthetic compounds were disclosed as using natural products [1,2,3]. In the particular case of cancer, the amount of natural product-based drugs rises up to 55%.

**Figure 1:**
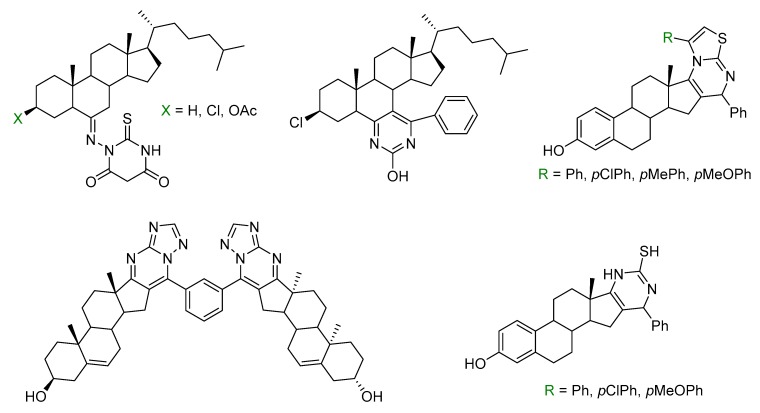
Illustrative biologically active steroidal pyrimidines.

When considering the chemical structure of small-molecule approved drugs, 59% of them contain a nitrogen heterocycle [4]. From this subset, six-membered rings (59%) are the most common, followed by five-membered (39%) and fused rings (14%). In particular, pyrimidine approved drugs are used for the treatment of three main disease classes: anti-infective, cardiovascular, and oncological. It has been reported that the conjugation of a pyrimidine moiety with a steroidal framework resulted in a synergistic effect of both biologically active molecular scaffolds providing new compounds with antiproliferative [5,6,7,8,9] (Figure 1), antioxidant [10], anti-Alzheimer [11], or antibacterial properties [12].

Based on the beneficial importance of these hybrid scaffolds, we moved our attention to the synthesis of steroidal pyrimidines in order to look for new antiproliferative compounds. We have previously reported some monomeric and dimeric steroidal triazolopyrimidine derivatives [13] and a family of steroidal spiro heterocycles from *trans*-androsterone and estrone bearing oxazolidin-2-one and 2-aminooxazoline motifs at C-17 [14]. A remarkable antiproliferative activity was found for some of the estrone derivatives with GI_50_ values at the low micromolar level.

Herein, we report the synthesis of six new pregnenolone and diosgenine-based pyrimidines attached or fused at C-17 and their evaluation as antiproliferative agents.

## 2. Results and Discussion

### 2.1. Chemistry

This pyrimidine formation includes 1,4-addition of 1,3-binucleophilic agents to an enone and the subsequent intramolecular addition of an NH_2_ group to ketone followed by elimination of H_2_O.

**Scheme 1:**
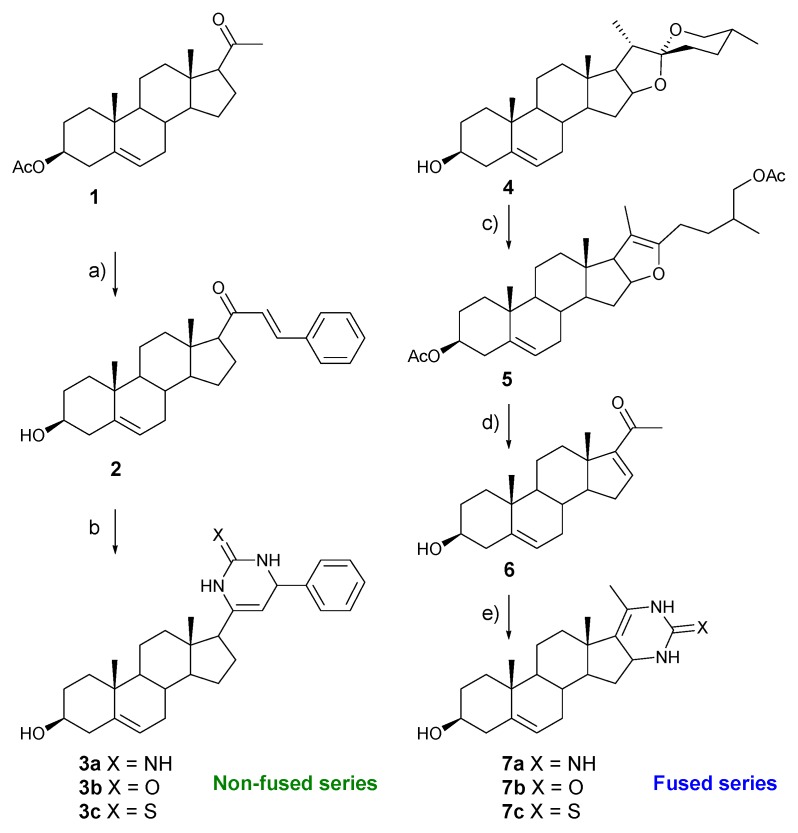
Reagent and conditions (**a**) PhCHO, KOH, EtOH, rt, 98%; (**b**) *t*-BuOK (THF 1M solution), BuOH, guanidine, urea, or thiourea, 56–68%; (**c**) BF_3_·Et_2_O, ATFAA, CH_2_Cl_2_, rt, 93%; (**d**) CrO_3_, AcOH, DCM, H_2_O, 0 °C to rt, 82%; and (**e**) KOH, EtOH, guanidine, urea, or thiourea, reflux, 52–65%.

We selected guanidine, urea, and thiourea as 1,3-binucleophilic agents and two related pregnenolone frameworks in order to prepare two series of steroidal pyrimidines, namely non-fused and fused pyrimidine steroids. The synthetic pathway is outlined in Scheme 1. The synthesis of the non-fused pyrimidine steroids (**3a**–**c**) was started from commercially available pregnenolone acetate (**1**). The acetate group was hydrolyzed under standard basic conditions and in situ the ketone was converted into the desired α,β-unsaturated ketone **2** through a Claisen–Schmidt condensation with benzaldehyde under basic conditions [15].

Finally, the cycloaddition reaction of **2** with guanidine, urea, or thiourea, respectively, was carried out. For the basic catalysis ethanolic solutions of KOH at diverse concentrations were essayed; however, the reaction did not take place.

Subsequently, changing the basic conditions to *t*-BuONa/BuOH afforded the non-fused pyrimidine steroids **3a**–**c** in 56–68% yield. Otherwise, for the synthesis of fused pyrimidine steroids **7a**–**c**, commercially available diosgenin **4** was transformed into the pseudosapogenin **5** through a selective F ring cleavage [16]. Then, the oxidative rupture of the intermediate **5** afforded the α,β-unsaturated ketone **6**. Finally, refluxing ketone **6** with guanidine, urea, or thiourea, respectively, in alcoholic solution of potassium hydroxide led to the fused pyrimidine steroids **7a**–**c** in 52–65% yield. Table 1 summarizes the results of the cycloaddition reaction of α,β-unsaturated ketones **2** and **6**. Reaction times were in the range 7–14 h, while yields were modest and ranging from 52% to 68%. These results pointed out that the reactivity of the 1,3-binucleophile was not affected by the electronegativity of the exocyclic heteroatom.

### 2.2. Antiproliferative Activity

We studied the antiproliferative activity of α,β-unsaturated ketones (**2** and **6**) and steroidal pyrimidines (**3** and **7**) against human cell lines following the guidelines of the National Cancer Institute [17]. For the tests we selected five human solid tumor cell lines from diverse origins and one human fibroblast cell line as a model of non-tumor cells. These cell lines were chosen as representative of drug sensitive (A549, HBL-100, and HeLa) and drug resistant (T-47D and WiDr) monolayer cultures based on previous studies. The steroidal anticancer drugs abiraterone and galeterone were also tested for comparison purposes. Table 2 shows the results expressed as 50% growth inhibition (GI_50_). Compound **7a** was not tested due to poor solubility under protocol conditions. Overall, all tested compounds (**2**, **3a**–**c**, **6**, and **7b**–**c**, compound **7a** was not tested due to poor solubility under protocol conditions) showed antiproliferative activity against all the tumor cell lines. They also displayed a good selectivity towards the human fibroblast cells (healthy cells). The potency of the compounds is comparable to that of abiraterone and galeterone, with GI_50_ values in the μM range. The most potent compound of the series is **7c**, a fused steroidal pyrimidinethione, which showed GI_50_ values in the range 2.6–3.7 μM against all tumor cells and is harmless for fibroblasts (GI_50_ > 100 μM). This is a relevant finding, since our compounds are non-toxic to healthy cells, whilst the steroidal anticancer drugs inhibit the growth of fibroblasts at GI_50_ values as low as 4.5–5 µM.

**Figure 2:**
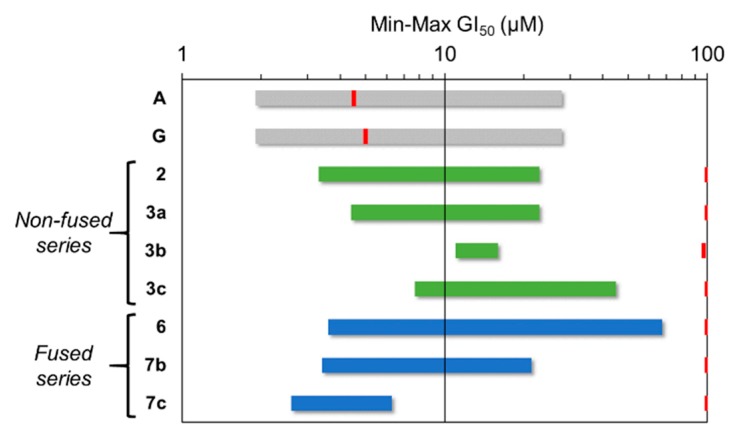
GI_50_ range plot against tested human solid tumor cell lines (Abiraterone, A; galeterone, G: gray; non-fused pyrimidine steroids: green; fused pyrimidine steroids: blue) and GI_50_ against human fibroblasts (red).

According to the GI_50_ values (Table 2) and the GI_50_ range plot (Figure 2), we can infer some structure activity relationships. The α,β-unsaturated ketone derivative **2** is more active than its non-fused derivatives **3a**–**c**. We speculate that the presence of the conjugated double bond in **2** favors the activity. For the non-fused series, the trend in antiproliferative activity in terms of the exocyclic heteroatom is N (**3a**) > O (**3b**) > S (**3c**). Interestingly, the antiproliferative effects observed for the fused series move in the opposite direction. Thus, α,β-unsaturated ketone **6** is the least active compound, and the favored exocyclic heteroatom is sulfur (**7c** > **7b**).

### 2.3. P-Glycoprotein Assay

Next, we studied if the compounds could be substrates for P-glycoprotein 1 (P-gp). P-gp is an important protein of the cell membrane that pumps xenobiotics out of cells. One of the resistance mechanisms to anticancer drugs involves the overexpression of P-gp by cancer cells, rendering these cancers multidrug resistant. Therefore, it is relevant to know whether compounds are substrates for P-gp or not [18]. As a model to test the effect of P-gp overexpression in our compounds, we used a cell line-based assay. We selected one wild type human lung cancer cell line (SW1573) and its P-gp overexpressing variant (SW1573/PGP) [19]. In this test, cells were exposed to the compounds for 48 h in the absence or the presence of 10 µM verapamil, a well-known P-gp inhibitor [20].

Table 3 shows the results expressed as GI_50_ values and the ratio of GI_50_ values in the P-gp overexpressing, as well as the wild type cell line, defined as the resistance factor (Rf). Overall, the results indicate that the α,β-unsaturated ketones **2** and **6**, and the steroidal pyrimidines **3** and **7**, are not substrates for P-gp. 

## 3. Materials and Methods 

### 3.1. Chemistry

#### 3.1.1. General Methods

Melting points were measured by the open capillary tube method on a Melt-temp apparatus and were not corrected. Optical rotations were measured on a Jasco P-2000 polarimeter (Jasco Inc, Easton, MD, USA). IR spectra were acquired on a Cary 630 FTIR spectrophotometer ($\bar{v}$_max_, cm^−1^) (Agilent, Santa Clara, CA, USA). NMR spectra were recorded on a Bruker Ascend 500 MHz instrument (Bruker BioSpin, Rheinstetten, Germany). Chemical shifts are reported in ppm (δ) and spectra were referenced to the residual protonated solvents: CDCl_3_ (7.26 and 77.16 ppm) and CD_3_OD (3.31 and 49.0 ppm) for ^1^H and ^13^C NMR, respectively. Coupling constants (*J*) are expressed in Hertz (Hz) and ^1^H-NMR data are reported as s (singlet), d (doublet), t (triplet), brs (broad singlet), and m (multiplet). ^1^H and ^13^C signals were assigned using 1D and 2D NMR experiments (DEPT, COSY, HSQC, HMBC, and NOESY). High resolution mass spectra were obtained by EI, ESI, and FAB using a Hewlett Packard 5989 A spectrometer coupled to a Hewlett Packard 5990 II gas chromatographer, a Micromass AutoSpec-Q, and a JEOL JMS-700 MStation mass spectrometer. Column chromatography was performed using Merck silica gel 60 (230–400 mesh). TLC was performed using aluminum pre-coated silica gel plates 60 F_254_; spots were visualized by UV light and charring with 5% H_2_SO_4_ (aq), 10% vanillin in EtOH containing 1% of H_2_SO_4_, or with 0.1% ninhydrin in EtOH.

#### 3.1.2. (*E*)-21-benzyliden-3β-hydroxypregn-5-en-20-one (**2**) 

To a solution of **1** (0.35 g, 1 mmol) in EtOH (5 mL) was added benzaldehyde (0.1 mL, 1 mmol) and KOH (10 mL, 10% in EtOH). The mixture was stirred at room temperature until the formation of the α,β-unsaturated ketone [15]. Then, the reaction mixture was extracted with CH_2_CI_2_ (3 × 25 mL). The combined organic phases were washed with brine (3 × 20 mL) and water (1 × 20 mL), and then dried over anhydrous MgSO_4_ and concentrated to dryness. The crude was purified by flash column chromatography (9:1 hexane-AcOEt) to afford product **2** as a white solid. Yield 98%; mp. 110–112 °C; [α]_D_^20^ −0.96 (c 1, CHCl_3_). IR (cm^−1^): 3320 (OH), 2939 (CH aliphatic), 2000 (CH aromatic), 1679 (C=O, α,β-unsaturated ketone), 1603 (C=C), 1053 (CO). ^1^H-NMR δ: 0.66 (s, 3H, CH_3_-18), 1.02 (s, 3H, CH_3_-19), 2.88 (t, 1H, *J* = 9.0 Hz, H-17), 3.55 (m, 1H, H-3), 5.39 (d, 1H, *J* = 5.2 Hz, H-6), 6.82 (d, 1H, *J* = 16.0 Hz, H-21), 7.40 (m, 3H, H-3′, H-4′, H-5′), 7.59 (m, 3H, H-2′, H-6′, H-22′).^13^C-NMR δ: 13.4 (C-18), 19.4 (C-19), 21.1 (C-11), 22.7 (C-15), 24.7 (C-16), 31.8 (C-7), 36.5 (C-2), 37.2 (C-8), 39.1 (C-1), 42.2 (C-10), 44.0 (C-12), 45.0 (C-4), 50.0 (C-13), 57.2 (C-9), 62.3 (C-14), 62.5 (C-17), 71.7 (C-3), 121.4 (C-6), 126.8 (C-21), 128.2 (C-2′and C-6′), 128.9 (C-3′and C-5′), 130.3 (C-4′), 134.8 (C-1′) 140.7 (C-5), 141.5 (C-22), 200.5 (C-20). HRMS-FAB: (*m*/*z*) [M + H]^+^ calculated for C_28_H_36_O_2_: 404.2715, found: 404.2786.

#### 3.1.3. General Procedure for the Preparation of **3a**–**c**

To a solution of **2** (0.1 g, 1 mmol) in dry BuOH (5 mL) was added the 1,3-binucleophile (thiourea, urea, or guanidine hydrochloride, 2.5 mmol) and *t*-BuOK solution 1 M in THF (0.99 mL, 8 mmol). The mixture was refluxed for 7–8 h and then extracted with CH_2_Cl_2_ (3 × 20 mL). The combined organic phases were washed with brine (1 × 20 mL), dried over MgSO_4_ and concentrated to dryness. The crude was purified by flash column chromatography (7:3 hexane-AcOEt) to afford **3a**–**c**.

*17β-(2′-imino-1′,3′,6′-trihydro-6′-phenylpyrimidin-4′-yl)-androst-5-en-3β-ol* (**3a**) White solid. Yield 68%; mp: 128 °C; [α]_D_^20^ −0.96 (c 0.11, CHCl_3_). IR (cm^−1^): 3334 (C=NH), 3320 (OH), 2939 (C-H aliphatic), 2010 (C-H aromatic), 1648 (C=N), 1044 (C-O, C-N), 800 (C-N). ^1^H-NMR δ: 0.57 (s, 3H, CH_3_-18), 0.98 (s, 3H, CH_3_-19), 3.52 (m, 1H, H-3), 5.27(d, 2H, *J* = 15 Hz, H-5′), 5.36 (d, 1H, *J* = 5.2 Hz, H-6), 6.79 (sa, 1H, H-3′), 6.59 (s, 1H, H-6′), 6.78 (brs, 1H, NH-1′), 6.87 (brs, 1H, NH-2′), 7.27 (brs, NH-3′), 7.44 (m, 2H, H-3′′and H-5′′), 7.46 (m, 2H, H-3′′, H-5′′), 7.95 (m, 2H, H-2′′ and H-6′′). ^13^C-NMR δ: 12.5 (C-18), 14.8 (C-11), 18.9 (C-19), 24.3 (C-15), 24.6 (C-2), 27.6 (C-4′), 31.5 (C-16), 32.1 (C-7), 33.6 (C-1), 36.1.(C-10), 36.8 (C-8), 37.6 (C-4), 41.8 (C-4), 44.5 (C-13), 49.8 (C-5), 56.4 (C-14), 57.5 (C-6′), 65.3 (C-17), 71.1 (C-3), 107.0 (C-5′) 120.1 (C-6), 127.0 (C-2′′), 128.6 (C-3′′ and C-6′′), 130.1 (C-4′′), 137.8 (C-5′), 137.9 (C-1′′), 131.5 (C-6′), 140.8 (C-4′), 140.9 (C-5), 162.5 (C-2′). HRMS-FAB: (*m*/*z*) [M + H]^+^ calculated C_29_H_39_N_3_O: 444.1015, found: 444.3015.

*17β-(1′,3′,6′-trihydro-2′-oxo-6′-phenylpyrimidin-4′-yl)-androst-5-en-3β-ol* (**3b**) White solid. Yield 56%; mp: 108-110 °C; [α]_D_^20^ −0.96 (c 0.11, CHCl_3_). IR (cm^−1^): 3320 (OH), 2929 (C-H aliphatic), 2010 (C-H aromatic), 1643 (C=O), 1047 (C-O), 771 (C-N). ^1^H-NMR δ: 0.74 (s, 3H, CH_3_-18), 0.82 (s, 3H, CH_3_-19), 3.56 (m, 1H, H-3), 5.35 (d, 1H, *J* = 5.2 Hz, H-6), 6.37 (d, 1H, *J* = 16 Hz, H-5′), 6.59 (d, 1H, *J* = 16 Hz, H-6′), 7.18 (d, 2H, *J* = 8 Hz, H-2′′ and H-6′′), 7.24 (m, 1H and H-4′), 7.31 (m, 2H, H-3′′ and H5′′). ^13^C-NMR δ: 12.3 (C-19), 13.6 (C-18), 15.9 (C-11), 21.6 (C-15), 23.5 (C-2), 27.6 (C-4′), 28.5 (C-16), 31.6 (C-7), 33.1 (C-1), 35.1.(C-10), 35.9 (C-8), 36.6 (C-4), 37.8 (C-12), 38.3 (C-13), 44.2 (C-5), 49.7 (C-14), 53.6 (C-6′), 60.6 (C-17), 71.6 (C-3), 120.5 (C-6), 126.1 (C-2′′ and C-6′′), 127.6 (C-4′′), 128.9 (C-3′′ and C-5′′), 128.9 (C-5′), 131.5 (C-6′), 138.3 (C-1′′),138.3 (C-1′′), 140.1 (C-5), 174.6.5 (C-2′). HRMS-FAB: (*m*/*z*) [M + H]^+^ calculated C_29_H_38_N_2_O_2_: 446.2933, found: 446.6350.

*17β-(1′,3′,6′-trihydro-6′-phenyl-2′-thioxopyrimidin-4′-yl)-androst-5-en-3β-ol* (**3c**) White solid. Yield 62%; mp: 208–210 °C; [α]_D_^20^ −37.99 (c 0.11, CHCl_3_). IR (cm^−1^): 3324 (OH), 2939 (C-H aliphatic), 2015 (C-H aromatic), 1059 (C=S), 755 (C-N). ^1^H-NMR δ: 0.55 (s, 3H, CH_3_-18), 0.93 (s, 3H, CH_3_-19), 3.46 (m, 1H, H-3), 4.67 (sa, 1H, H-5′), 5.10 (sa, 1H, H-6′), 5.26 (d, 1H, *J* = 5.2 Hz, H-6) 6.63 (sa, 1H, N-H3′), 7.00 (sa, 1H, N-H1′), 7.22 (m, 5H, H-aromatic). ^13^C-NMR δ: 13.0 (C-18), 19.4 (C-19), 19.8 (C-11), 21.1 (C-15), 24.0 (C-2), 24.0 (C-4′), 29.9 (C-16), 31.8 (C-7), 32.3 (C-1), 36.7 (C-15), 37.4 (C-10), 36.5 (C-8), 37.1 (C-4), 38.4 (C-12), 42.1 (C-13), 49.8 (C-9), 55.8 (C-17), 56.9 (C-6′), 71.6 (C-3), 99.9 (C-5′), 121.2 (C-6), 126.8 (C-2′′and C-6′′), 134.2 (C-4′), 129.0 (C-3′and C5′′), 134.2 (C-1′′), 142.8 (C-4′′), 140.9 (C-5), 174.3 (C-2′). HRMS-FAB: (*m*/*z*) [M + H]^+^ calculated C_29_H_38_N_2_OS: 462.2705, found: 462.6960.

#### 3.1.4. (25R)-furosta-5,20(22)-diene-3β,26-diyl Diacetate (**5**)

To a solution of diosgenin (**4**, 0.83 g, 2.0 mmol) in CH_2_Cl_2_ (10 mL) were sequentially added BF_3_·OEt_2_ (1.0 mL, 7.96 mmol) and freshly prepared acetic trifluoracetic anhydride (4.7 mL, 21.8 mmol). The mixture was magnetically stirred at room temperature and monitored by TLC until complete conversion of the starting material (approximately 5 min). The mixture was poured over ground ice (20 g) and vigorously shaken. The crude product was washed with saturated solution of NaHCO_3_ (3 × 20 mL) and brine (3 × 20 mL). The organic phase was dried using anhydrous MgSO_4_ and concentrated to dryness. The crude was purified by flash chromatography (8:2 hexane-AcOEt) to give **5** as a white solid. Yield 93%; mp: 59–60 °C; [α]_D_^20^ −27.4 (c 1.09, CHCl_3_). IR (cm^−1^): 2941 (C-H aliphatic), 1727 (C=O ester), 1661 (C=C), 1231 (O-C-O), 1027 (C-O). ^1^H-NMR δ: 0.69 (s, 3H, CH_3_-18), 0.94 (d, 3H, *J* = 7.2 Hz, CH_3_-27), 1.03 (s, 3H, CH_3_-19), 1.58 (s, 3H, CH_3_-21), 2.02 (s, 3H, CH_3_COO-3), 2.04 (s, 3H, CH_3_COO-26), 2.47 (d, 1H, *J* = 8.5 Hz, H-17), 3.87 (dd, 1H, *J_26α,26β_* = 10.6 Hz, *J*_26*α*,25_ = 5.6 Hz, H-26α), 3.93 (dd, 1H, *J_26β,26α_* = 10.6 Hz, *J*_26β,25_ = 5.6 Hz, H-26β), 4.58 (m, 1H, H-3), 4.72 (m, 1H, H-16), 5.36 (d, 1H, *J* = 4 Hz, H-6). ^13^C-NMR δ: 11.7 (C-21), 14.0 (C-18), 16.8 (C-27), 9.4 (C-19), 21.0 (C-11), 21.5 (CH_3_COO-3), 23.2 (C-23), 27.7 (C-2), 30.8 (C-24), 31.2 (C-8), 32.1 (C-7), 32.1 (C-25), 34.2 (C-15), 36.6 (C-10), 37.0 (C-1), 38.1 (C-4), 39.4 (C-12), 43.2 (C-13), 49.9 (C-9), 54.9 (C-14), 64.1 (C-17), 69.7 (C-26), 73.7 (C-3), 84.2 (C-16), 103.6 (C-20), 122.1 (C-6),139.4 (C-5), 151.1 (C-22), 170.2 (CH_3_COO-3), 171.2 (CH_3_COO-26). HRMS-FAB: (*m*/*z*) [M + H]^+^ calculated C_31_H_46_O_5_: 498.33, found: 498.69.

#### 3.1.5. 20-Oxopregna-5,16-dien-3β-yl Acetate (**6**)

A solution of pseudodiosgenin diacetate (**5**) (2.0 g, 4 mmol) in CH_2_CI_2_ (8 mL), acetic acid (8 mL) and water (1 mL) was cooled to 0 °C. Then, a solution of CrO_3_ (0.84 g, 8.5 mmol) in water (1.2 mL) and acetic acid (0.4 mL) was added dropwise. The mixture was magnetically stirred at room temperature and monitored by TLC until disappearance of the starting material (4 h). The mixture was extracted with CH_2_Cl_2_ (3 × 20 mL) and then treated with a saturated solution of NaCl in stirred for 15 min. The mixture was extracted with CH_2_Cl_2_ (2 × 20 mL), the organic phases were washed with water (3 × 20 mL), dried over MgSO_4_ and concentrated to dryness. The crude was maintained under reflux in acetic acid and monitored with TLC until observation of a more polar compound. After completion of the reaction, water was added and neutralized with a saturated solution of NaHCO_3_. The organic phase was dried over MgSO_4_ and concentrated to dryness. The crude was purified by flash column chromatography (9:1 hexane-AcOEt) to give compound **6** as a white solid. Yield 82%; mp: 108–110 °C; [α]_D_^20^ +0.86 (c 1.02, CHCl_3_). IR (cm^−1^): 3320 (OH), 2939 (C-H aliphatic), 1700 (C=O α,β-unsaturated ketone), 1027 (C-O). ^1^H-NMR δ: 0.92 (s, 3H, CH_3_-18), 1.06 (s, 3H, CH_3_-19), 2.04 (s, 3H, CH_3_COO-3) 2.27 (s, 3H, CH_3_-21), 4.55 (m, 1H, H-3), 5.33 (d, 1H, *J* = 4 Hz, H-6), 6.67 (d, 1H, *J* = 4 Hz, H-16). ^13^C-NMR δ: 15.6 (C-18), 19.1 (C-19), 20.5 (CH_3_COO-3), 21.4 (C-11), 27.1 (C-21), 27.6 (C-2), 30.0 (C-7), 31.4 (C-15), 32.2 (C-12), 34.5 (C-8), 36.7 (C-4), 46.0 (C-13), 50.2 (C-9), 56.2 (C-14), 73.6 (C-3), 121.9 (C-6), 140.0 (C-5), 144.4 (C-16), 155.2 (C-17), 170.5 (CH_3_COO-3), 196.8 (C-20). HRMS-FAB: (*m*/*z*) [M + H]^+^ calculated C_21_H_30_O_2_: 356.2351, found: 356.5060.

#### 3.1.6. General Procedure for the Preparation of **7a**–**c**

Compound **6** (0.2 g, 0.63 mmol) was dissolved in an alcoholic solution of KOH (1%) and 1,3-binucleophile was added (thiourea, urea, or guanidine hydrochloride, 1.2 mmol). The mixture was maintained under reflux for 8–14 h and monitored by TLC until disappearance of the starting material. Then, grinded ice was added and extracted with CH_2_CI_2_ (3 × 20 mL). The combined organic phase was washed with brine (2 × 20 mL) and water (1 × 20 mL), dried over MgSO_4_ and concentrated to dryness under vacuum. The crude was purified by flash column chromatography (7:3 hexane-AcOEt).

*1′,3′-dihydro-2′-imino- 4′-methylpyrimidin-(6′,5′:16,17)-androst-5-en-3β-ol* (**7a**) White solid. Yield 58%; mp: 108–110 °C; [α]_D_^20^ −0.96 (c 0.11, CHCl_3_). IR (cm^−1^): 3320 (OH), 3187 (C-N), 2939 (C-H), 1709 (C=N), 1042 (C-O), 750 (C-N). ^1^H-NMR δ: 0.93 (s, 3H, CH_3_-18), 1.18 (s, 3H, CH_3_-19), 1.91 (s, 3H, CH_3_-4′), 3.58 (m, 1H, H-3), 3.98 (m, 1H, H-16), 5.30 (d, 1H, *J* = 4 Hz, H-6), 6.14 (s, 1H, NH-1′), 6.81 (s, 1H, NH-3′), 7.29 (sa, 1H, NH-2′). ^13^C RMN δ: 14.0 (C-4′′), 16.9 (C-19), 19.2 (C-18), 20.8 (C-11), 29.3 (C-1), 29.6 (C-7), 31.4 (C-8), 33.9. (C-15), 35.9 (C-12), 36.6 (C-10), 41.8 (C-4), 42.1 (C-12), 43.5 (C-13), 50.2 (C-9), 53.2 (C-16), 55.2 (C-14), 71.3 (C-3), 120,8 (C-6), 120.8.5 (C-4′), 132.2 (C-17), 141.2 (C-5), 174.5 (C-2′). HRMS-FAB: (*m*/*z*) [M + H]^+^ calculated C_22_H_32_N_2_OS: 355.5624, found: 356.5260.

*1′,3′-dihydro-4′-methyl-2′-oxopyrimidin-(6′,5′:16,17)-androst-5-en-3β-ol* (**7b**) White solid. Yield 52%; mp: 163–164 °C; [α]_D_^20^ −0.96 (c 0.11, CHCl_3_). IR (cm^−1^): 3307 (OH), 2927 (C-H aliphatic), 1647 (C=O), 1047 (C-O), 771 (C-N). ^1^H-NMR δ: 0.85 (s, 3H, CH_3_-18), 0.97 (s, 3H, CH_3_-19), 1.78 (s, 3H, CH_3_-4′), 3.45 (m, 1H, H-3), 4.35 (d, 1H, *J* = 8 Hz, H-16), 5.35 (d, 1H, *J* = 4 Hz, H-6), 5.75 (s, 1H, NH-1′), 6.79 (s, 1H, NH-3′). ^13^C-NMR δ: 15.2 (C-4′), 16.4 (C-19), 19.4 (C-18), 20,4 (C-11), 31.1 (C-1), 31.2 (C-7), 31.6 (C-8), 32.6 (C-15), 35.5 (C-12), 36.2 (C-10), 36.5 (C-4), 36.8 (C-12), 41.6 (C-13), 49.7 (C-9), 52.3 (C-16), 54.5 (C-14), 65.6 (C-3), 120.6 (C-6), 121.4 (C-4′), 123.0 (C-17), 140.8 (C-5), 157.4 (C-2′). HRMS-FAB: (*m*/*z*) [M + H]^+^ calculated C_22_H_32_N_2_OS: 356.2264, found: 356.5110.

*1′,3′-dihydro-4′-methyl-2′-thionepyrimidin-(6′,5′:16, 17)-androst-5-en-3β-ol* (7c) White solid. Yield 65%; mp: 208–210 °C; [α]_D_^20^ −37.99 (c 1.02, CHCl_3_). IR (cm^−1^): 3320 (OH), 2939 (C-H aliphatic), 1201 (C=S), 1042 (C-O), 750 (C-N). ^1^H-NMR δ: 0.89 (s, 3H, CH_3_-18), 1.02 (s, 3H, CH_3_-19), 1.87 (s, 3H, CH_3_-4′), 3.46 (m, 1H, H-3), 4.20 (d, 1H, *J* = 8 Hz, H-16), 5.28 (d, 1H, *J* = 4 Hz, H-6), 6.87 (s, 1H, H-1′), 7.87 (s, 1H, H-3′). ^13^C-NMR δ: 15.2 (C-4′), 16.4 (C-19), 19.5 (C-18), 20.6 (C-11), 31.2 (C-1), 31.3 (C-7), 31.4 (C-8), 32.4 (C-15), 35.8 (C-12), 36.6 (C-10), 37.0 (C-4), 42.1 (C-12), 43.7 (C-13), 49.5 (C-9), 53.2 (C-16), 54.5 (C-14), 71.3 (C-3), 120,8 (C-6), 121.5 (C-4′), 122.6 (C-17), 140,8 (C-5), 176.18 (C-2′). HRMS-FAB: (*m*/*z*) [M + H]^+^ calculated C_22_H_32_N_2_OS: 372.2235, found: 372.2269.

1H- and 13C-NMR charts are available in Supplementary Materials.

### 3.2. Biological Tests

#### 3.2.1. Cell Lines and Growth Conditions

The human solid tumor cell lines A549 (non-small cell lung), HBL-100 (breast), and HeLa (cervix), SW1573 (non-small cell lung), and its P-gp overexpressing variant (SW1573/Pgp), T-47D (breast), and WiDr (colon) were used in this study. These cell lines used in this study were kindly provided by Dr. Godefridus J. Peters (Cancer Center Amsterdam, Vrije Universiteit, Amsterdam, The Netherlands). BJ-hTERT human fibroblast cells were given by Dr. Raimundo Freire (Universidad de La Laguna, Canary Islands). All cells were grown in RPMI 1640 supplemented with 1 mM glutamine, 5% FBS and antibiotics. Cells were grown at 37 °C in a humidified atmosphere of 5% CO_2_ and maintained at low passage.

#### 3.2.2. Growth Inhibition Assays

The antiproliferative activity was tested in vitro against human cancer cells using the protocol of the National Cancer Institute (NCI) of the USA16 with minor modifications [21]. The following densities (cells per well) were used: 2500 (A549, HBL-100, HeLa and SW1573), 5000 (T-47D, WiDr and SW1573/PGP), and 7000 (BJ-hTERT). Samples for testing were dissolved initially in DMSO at 40 mM, i.e., 400 times the maximum test concentration. Abiraterone and galeterone were used as reference drugs for antiproliferative tests, verapamil was used as a P-gp transport inhibitor in SW1573/Pgp experiments, and their stock solutions were prepared in DMSO at 40 mM. Paclitaxel and vinblastine were used as positive controls in SW1573/Pgp experiments and were initially dissolved in DMSO at 4 mM. For each test compound, the cells were exposed to serial decimal dilutions in the range of 0.001–100 μM for a period of 48 h. Cell culture medium containing verapamil was prepared by adding the final concentration of verapamil of 10 μM. For each product GI_50_ values were calculated according to the NCI formulas.

## 4. Conclusions

Pregnenolone acetate (**1**) and diosgenin (**4**) were efficiently transformed into α,β-unsaturated ketones **2** and **6**, respectively. These intermediates were treated with 1,3-binucleophiles (guanidine, urea or thiourea) to produce steroidal pyrimidines **3** and **7**, respectively. Antiproliferative activities were found for all tested compounds with good selectivity towards cancer cells. Furthermore, these compounds did not act as P-glycoprotein substrates, thus avoiding the rise of drug resistance. Overall, fused steroidal pyrimidinethione **7c** could be selected as the lead for further testing due to its strong antiproliferative activities within the low micromolar range. In summary, we have shown that the natural products reservoir represents a fast and reliable source for chemical scaffolds that can be transformed in a few reaction steps into pharmacologically relevant chemical entities. This work definitively establishes a versatile platform for the synthesis of a number of bioactive steroidal pyrimidines with the possibility of even further accessing the unnatural analogues of these steroidal pyrimidines using similar chemistry. The combined findings indicate that steroidal pyrimidines offer a source of novel bioactive molecules that could be used in the treatment of human diseases.

## Figures and Tables

**Table 1:** Outcome of the cycloaddition reaction of ketones **2** and **6**.

Series	Compound	X	Time (h)	Yield (%)
Non-fused	**3a**	N	7	68
	**3b**	O	12	56
	**3c**	S	10	62
Fused	**7a**	N	9	58
	**7b**	O	14	52
	**7c**	S	8	65

**Table 2:** Antiproliferative activity (GI_50_) against human solid tumor cells and human fibroblasts.^a^

Compound	Cell Line (Origin)					
	A549 (Lung)	HBL-100 (Breast)	HeLa (Cervix)	T-47D (Breast)	WiDr (Colon)	BJ-hTERT (Fibroblasts)
**2**	23 ± 1.6	19 ± 3.7	3.3 ± 0.3	9.9 ± 3.5	6.3 ± 0.5	>100
**3a**	4.4 ± 1.0	23 ± 2.8	6.1 ± 1.2	8.8 ± 2.2	8.3 ± 1.1	>100
**3b**	11 ± 2.9	16 ± 1.3	14 ± 3.6	15 ± 1.3	15 ± 3.5	97 ± 5.9
**3c**	7.7 ± 1.0	45 ± 7.9	11 ± 1.5	13 ± 2.3	9.2 ± 1.6	>100
**6**	26 ± 5.2	64 ± 1.6	3.6 ± 0.4	29 ± 4.5	37 ± 2.1	>100
**7b**	13 ± 3.7	15 ± 3.2	3.4 ± 0.2	15 ± 1.9	18 ± 1.8	>100
**7c**	3.4 ± 0.5	3.7 ± 1.0	2.6 ± 0.7	3.0 ± 0.5	3.1 ± 0.4	>100
Abiraterone	95 ± 0.8	>100	7.9 ± 0.5	24 ± 4.5	42 ± 7.7	4.5 ± 1.0
Galeterone	3.9 ± 1.3	10 ± 0.9	5.3 ± 0.4	2.1 ± 0.1	2.7 ± 0.2	5.0 ± 1.3

^a^ GI_50_ values are given in µM. Standard deviation was calculated from at least two independent experiments. Abiraterone and galeterone were used as reference drugs. Values in bold face represent the best antiproliferative data against tumor cell lines (GI_50_ < 10 µM).

**Table 3:** Antiproliferative activity (GI_50_) against SW1573 and SW1573/PGP cell lines.^a^

Compound	w/o Verapamil			w Verapamil		
	SW1573	SW1573/PGP	Rf	SW1573	SW1573/PGP	Rf
**2**	23 ± 0.2	29 ± 5.5	1.2	20 ± 2.9	30 ± 5.9	1.5
**3a**	6.9 ± 0.5	10 ± 4.2	1.5	8.1 ± 0.7	5.8 ± 0.1	0.7
**3b**	18 ± 0.9	34 ± 13	1.9	18 ± 3.4	28 ± 5.7	1.6
**3c**	12 ± 2.7	38 ± 6.1	3.2	14 ± 4.4	25 ± 1.1	1.8
**6**	72 ± 10	>100.0	1.4	71 ± 9.1	90 ± 19	1.3
**7b**	34 ± 4.7	49 ± 4.4	1.5	24 ± 3.0	33 ± 10	1.4
**7c**	9.4 ± 1.0	16 ± 1.3	1.7	8.1 ± 0.5	8.5 ± 1.8	1.1
PTX	1.5 ± 0.5	196 ± 53	128	1.6 ± 0.2	4.2 ± 0.9	2.6
VB	0.9 ± 0.3	2051 ± 682	2388	0.8 ± 0.2	1.0 ± 0.5	1.3

^a^ Paclitaxel (PTX) and vinblastine (VB) were used as reference drugs. GI_50_ values are given in µM for compounds **2**–**3** and **6**–**7**, and in nM for PTX and VB. Standard deviation was calculated from at least two independent experiments. Rf represents the ratio between GI_50_ values in SW1573/PGP and SW1573 cells.

## Supplementary Materials

The following are available online at https://www.mdpi.com/1420-3049/24/20/3676/s1 containing ^1^H- and ^13^C-NMR charts.

## References

[1] Newman D.J., Cragg G.M. (2016). Natural Products as Sources of New Drugs from 1981 to 2014. J. Nat. Prod..

[2] Carbone A., Parrino B., Cusimano M.G., Spanò V., Montalbano A., Barraja P., Schillaci D., Cirrincione G., Diana P., Cascioferro S. (2018). New thiazole nortopsentin analogues inhibit bacterial biofilm formation. Mar. Drugs.

[3] Spanò V., Attanzio A., Cascioferro S., Carbone A., Montalbano A., Barraja P., Tesoriere L., Cirrincione G., Diana P., Parrino B. (2016). Synthesis and antitumor activity of new thiazole nortopsentin analogs. Mar. Drugs.

[4] Vitaku E., Smith D.T., Njardarson J.T. (2014). Analysis of the structural diversity, substitution patterns, and frequency of nitrogen heterocycles among U.S. FDA approved pharmaceuticals. J. Med. Chem..

[5] Shamsuzzaman, Dar A.M., Yaseen Z., Alam K., Hussain A., Gatoo M.A. (2013). Steroidal pyrimidines; Synthesis, characterization, molecular docking studies with DNA and vitro cytotoxicity. J. Mol. Struct..

[6] Shamsuzzaman, Dar A.M., Tabassum S., Zaki M., Khan Y., Sohail A., Gatoo M.A. (2014). DNA binding, docking studies, artificial nuclease activity and in vitro cytotoxicity of newly synthesized steroidal 1H-pyrimidines. C. R. Chimie.

[7] Dar A.M., Rah B., Mir S., Nabi R., Shamsuzzaman, Gatoo M.A., Mashrai A., Khand Y. (2018). DNA binding, artificial nuclease activity and cytotoxic studies of newly synthesized steroidal pyrimidines. Int. J. Biol. Macromol..

[8] Mohareb R.M., Al-Omran F., Azzam R.A. (2014). Heterocyclic ring extension of estrone: Synthesis and cytotoxicity of fused pyran, pyrimidine and thiazole derivatives. Steroids.

[9] Yu B., Shi X.-J., Zheng Y.-F., Fang Y., Zhang E., Yu D.-Q., Liu H.-M. (2013). A novel [1,2,4] triazolo [1,5-a] pyrimidine-based phenyl-linked steroid dimer: Synthesis and its cytotoxic activity. Eur. J. Med. Chem..

[10] Ali A., Asif M., Alam P., Alam J.M., Sherwani A.M., Khan H.R., Ahmad S., Shamsuzzaman (2017). DFT/B3LYP calculations, in vitro cytotoxicity and antioxidant activities of steroidal pyrimidines and their interaction with HSA using molecular docking and multispectroscopic techniques. Bioorg. Chem..

[11] Abdalla M.M., Al-Omar M.A., Bhat M.A., Amr A.E., Al-Mohizea A.M. (2012). Steroidal pyrazolines evaluated as aromatase and quinone reductase-2-inhibitors for chemoprevention of cancer. Int. J. Biol. Macromol..

[12] Abdelhalim M.M., El-Saidi M.M.T., Rabie S.T., Elmegeed G.A. (2007). Synthesis of novel steroidal heterocyclic derivatives as antibacterial agents. Steroids.

[13] Arenas-González A., Mendez-Delgado L.A., Merino-Montiel P., Padrón J.M., Montiel-Smith S., Vega-Báez J.L., Meza-Reyes S. (2016). Synthesis of monomeric and dimeric steroids containing [1,2,4]triazolo[1,5-a]pyrimidines. Steroids.

[14] Romero-Hernández L.L., Merino-Montiel P., Meza-Reyes S., Vega-Baez J.L., López Ó., Padrón J.M., Montiel-Smith S. (2018). Synthesis of unprecedented steroidal spiro heterocycles as potential antiproliferative drugs. Eur. J. Med. Chem..

[15] Romero-López A., Montiel-Smith S., Meza-Reyes S., Merino-Montiel P., Vega-Baez J.L. (2014). Synthesis of steroidal derivatives containing substituted, fused and spiro pyrazolines. Steroids.

[16] Viñas-Bravo O., Martínez-Pascual R., Vega-Báez J.L., Gómez-Calvario V., Sandoval-Ramírez J., Montiel Smith S., Meza-Reyes S., López-De la Rosa A., Martínez-Montiel M., Reyes M. (2012). Rapid conversion of spirostans into furostan skeletons at room temperatura. Steroids.

[17] Monks A., Scudiero D., Skehan P., Shoemaker R., Paull K., Vistica D., Hose C., Langley J., Cronise P., Vaigro-Wolff A. (1991). Feasibility of a High-Flux Anticancer Drug Screen Using a Diverse Panel of Cultured Human Tumor Cell Lines. J. Natl. Cancer Inst..

[18] Chang C., Bahadduri P.M., Polli J.E., Swaan P.W., Ekins S. (2006). Rapid Identification of P-glycoprotein Substrates and Inhibitors. Drug Metab. Dispos..

[19] Bergman A.M., Pinedo H.M., Talianidis I., Veerman G., Loves W.J., Van der Wilt C.L., Peters G.J. (2003). Increased sensitivity to gemcitabine of P-glycoprotein and multidrug resistance-associated protein-overexpressing human cancer cell lines. Br. J. Cancer.

[20] Castaing M., Loiseau A., Cornish-Bowden A. (2007). Synergy between verapamil and other multidrug-resistance modulators in model membranes. J. Biosci..

[21] Miranda P.O., Padrón J.M., Padrón J.I., Villar J., Martín V.S. (2006). Prins-type synthesis and SAR study of cytotoxic alkyl chloro dihydropyrans. ChemMedChem.

