# Supplementary material for: Synthesis and Evaluation of Pyrimidine Steroids as Antiproliferative Agents
Source: Molecules. 2019 Oct 12;24(20):3676. doi: 10.3390/molecules24203676 (PMC6832952; doi:10.3390/molecules24203676)
Supplement: Supplementary file 1 [file molecules-24-03676-s001.pdf]

Supplementary Information

Synthesis and evaluation of pyrimidine steroids as antiproliferative agents

Alejandra Cortés-Percino[a], José Luis Vega-Báez[a], Anabel Romero-López[b], Adrián Puerta[c], Penélope Merino-Montiel[a], Socorro Meza-Reyes[a], José M. Padrón[c,]*, Sara Montiel-Smith[a]*

[a] *Facultad de Ciencias Químicas, Benemérita Universidad Autónoma de Puebla, Ciudad Universitaria, 72570 Puebla, Pue., Mexico. e-mail: maria.montiel@correo.buap.mx*

[b] *Instituto de Física "Luis Rivera Terrazas" Benemérita Universidad Autónoma de Puebla Ecocampus Valsequillo, 72960 San Pedro Zacachimalpa, Pue., Mexico*

[c] *BioLab, Instituto Universitario de Bio-Orgánica "Antonio González" (IUBO-AG), Centro de Investigaciones Biomédicas de Canarias (CIBICAN), Universidad de La Laguna, c/ Astrofísico Francisco Sánchez 2, 38206 La Laguna, Spain. e-mail: jmpadron@ull.es*

Table of contents

Figures S1. ^{1}H and ^{13}C NMR of compound **2**

Figures S2. ^{1}H and ^{13}C NMR of compound **3c**

Figures S3. ^{1}H and ^{13}C NMR of compound **6**

Figures S4. ^{1}H and ^{13}C NMR of compound **7a**

Figures S5. ^{1}H and ^{13}C NMR of compound **7b**

Figures S6. ^{1}H and ^{13}C NMR of compound **7c**

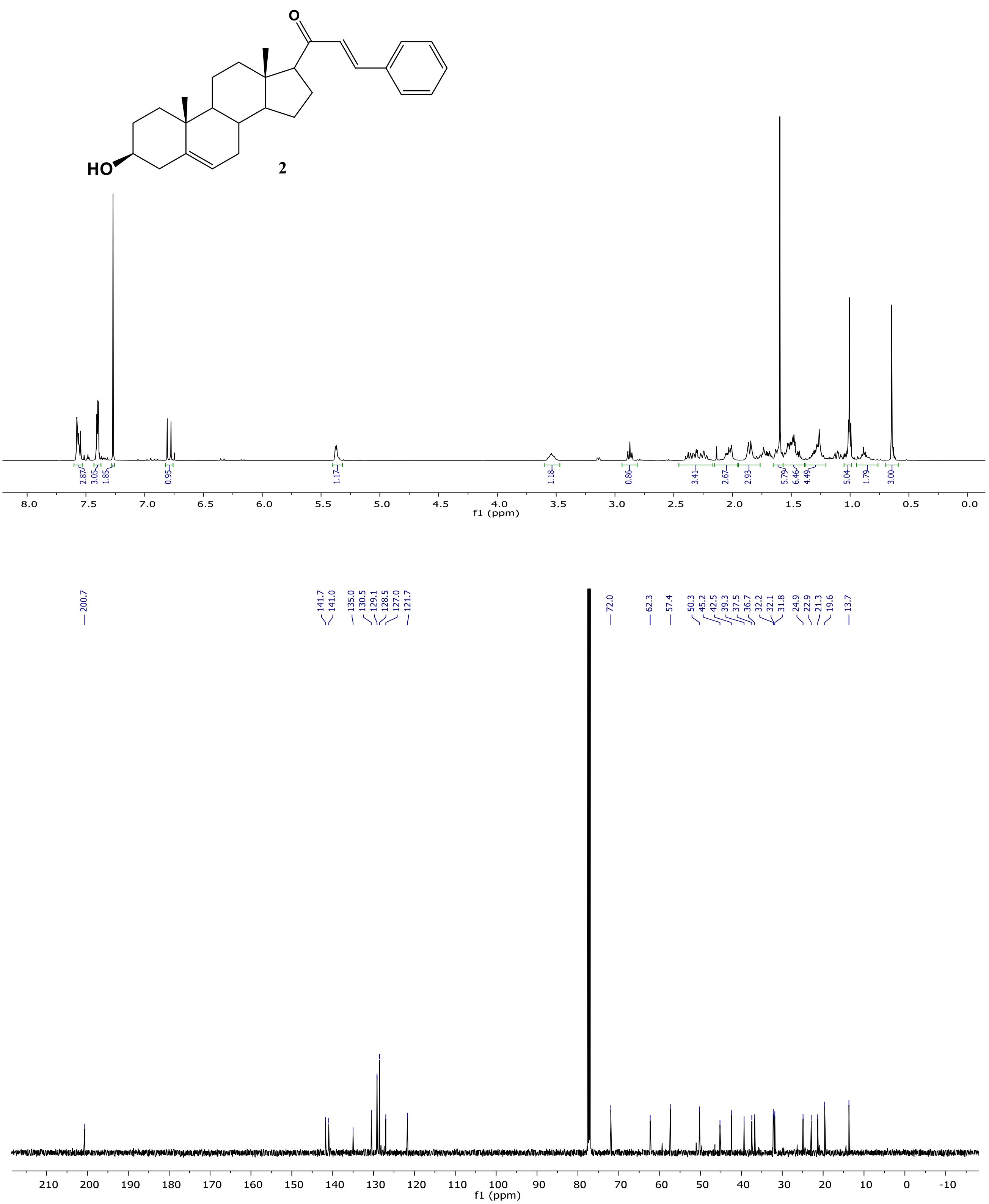

Figure S1. ^{1}H NMR (500 MHz, $CDCl_3$) and ^{13}C NMR (125 MHz, $CDCl_3$) of **2**.

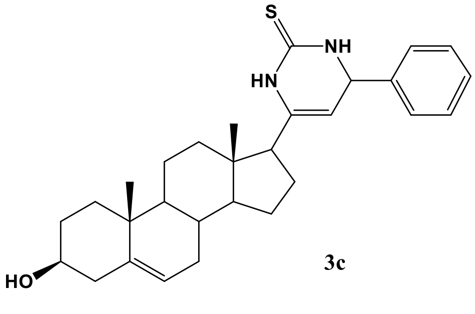

3c

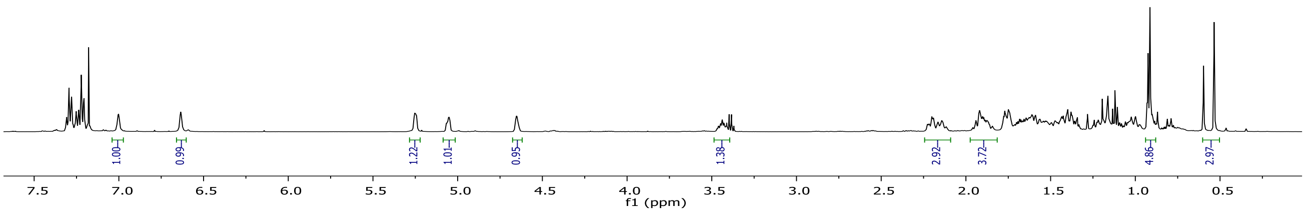

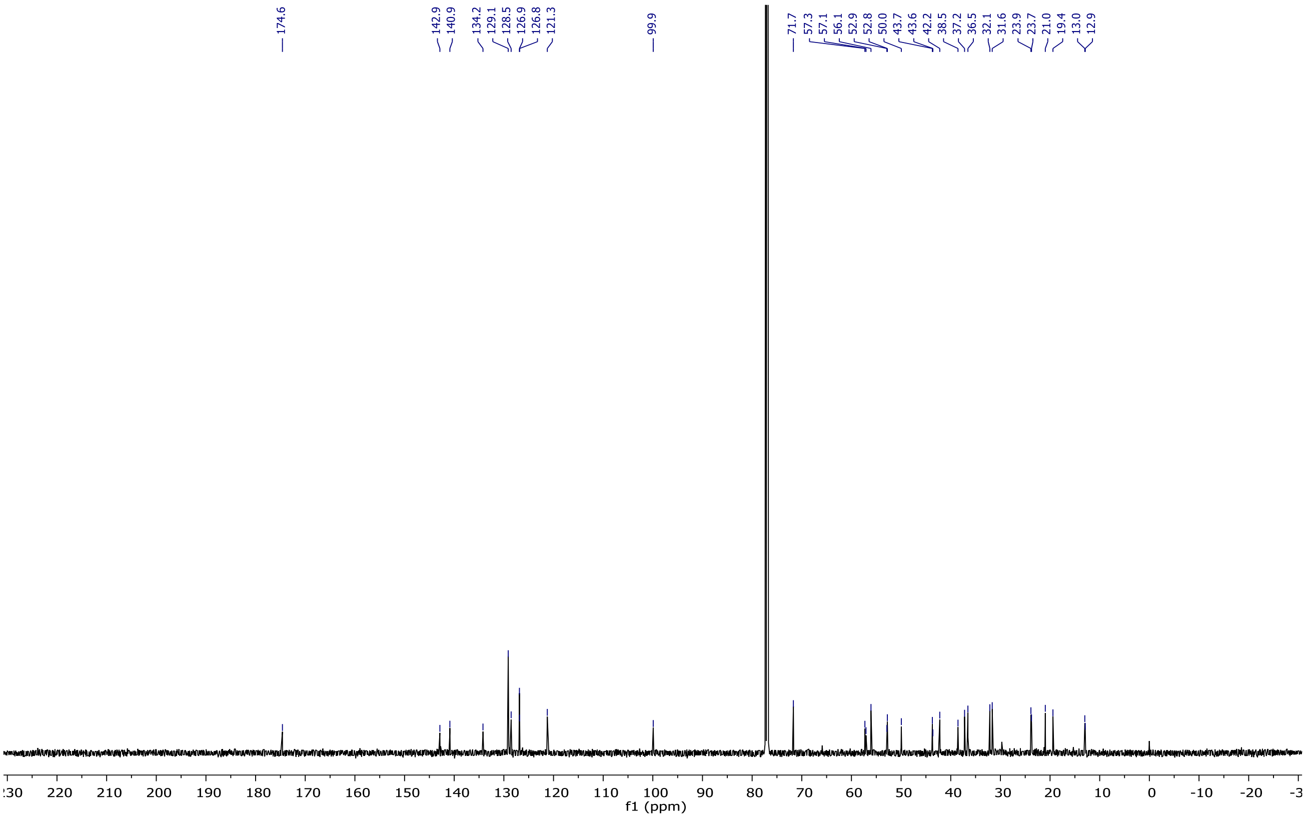

Figure S2. ^{1}H NMR (500 MHz, $CDCl_3$) and ^{13}C NMR (125 MHz, $CDCl_3$) of **3c**.

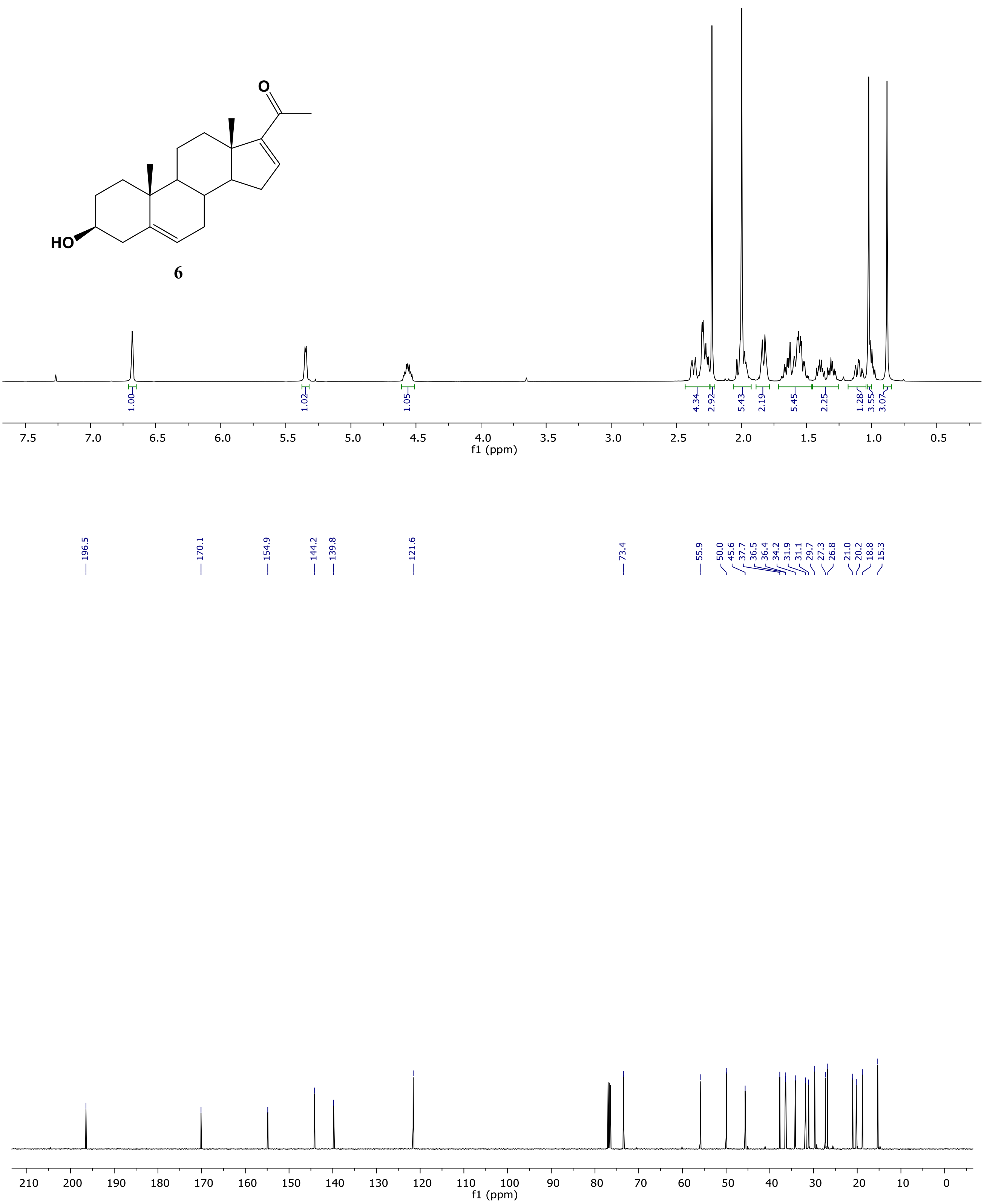

Figure S3. ^{1}H NMR (500 MHz, $CDCl_3$) and ^{13}C NMR (125 MHz, $CDCl_3$) of **6**.

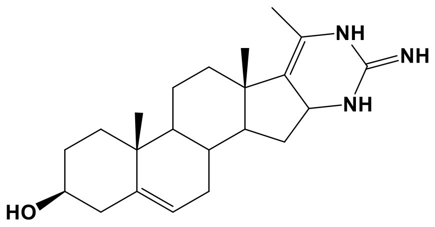

7a

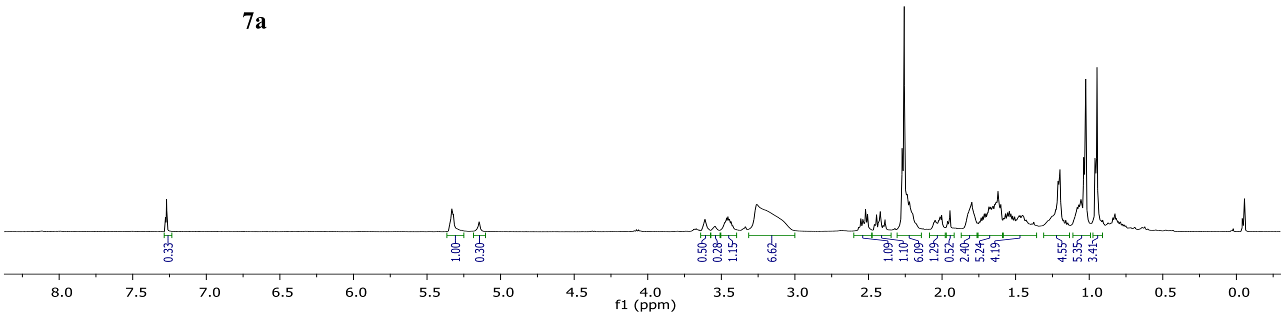

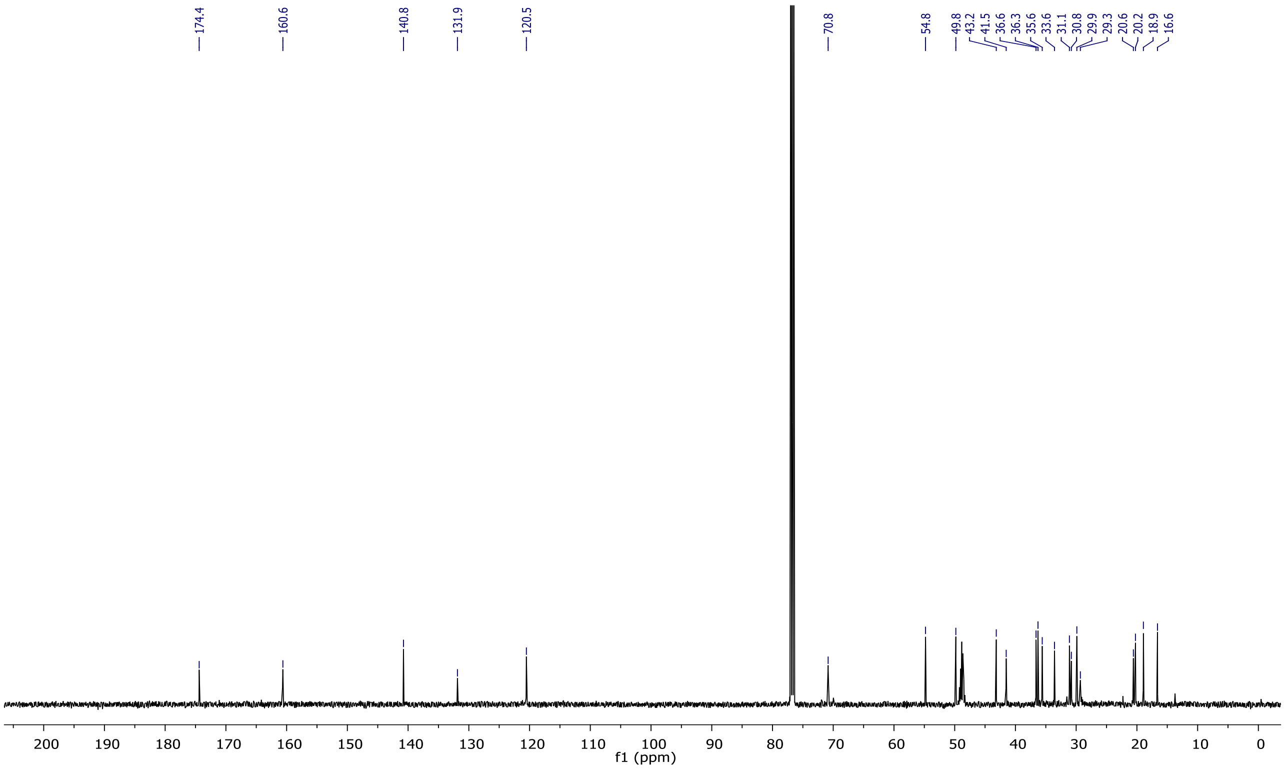

Figure S4. ^{1}H NMR (500 MHz, $CDCl_3$-MeOD) and ^{13}C NMR (125 MHz, $CDCl_3$-MeOD) of **7a**.

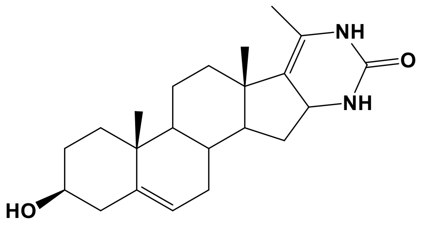

7b

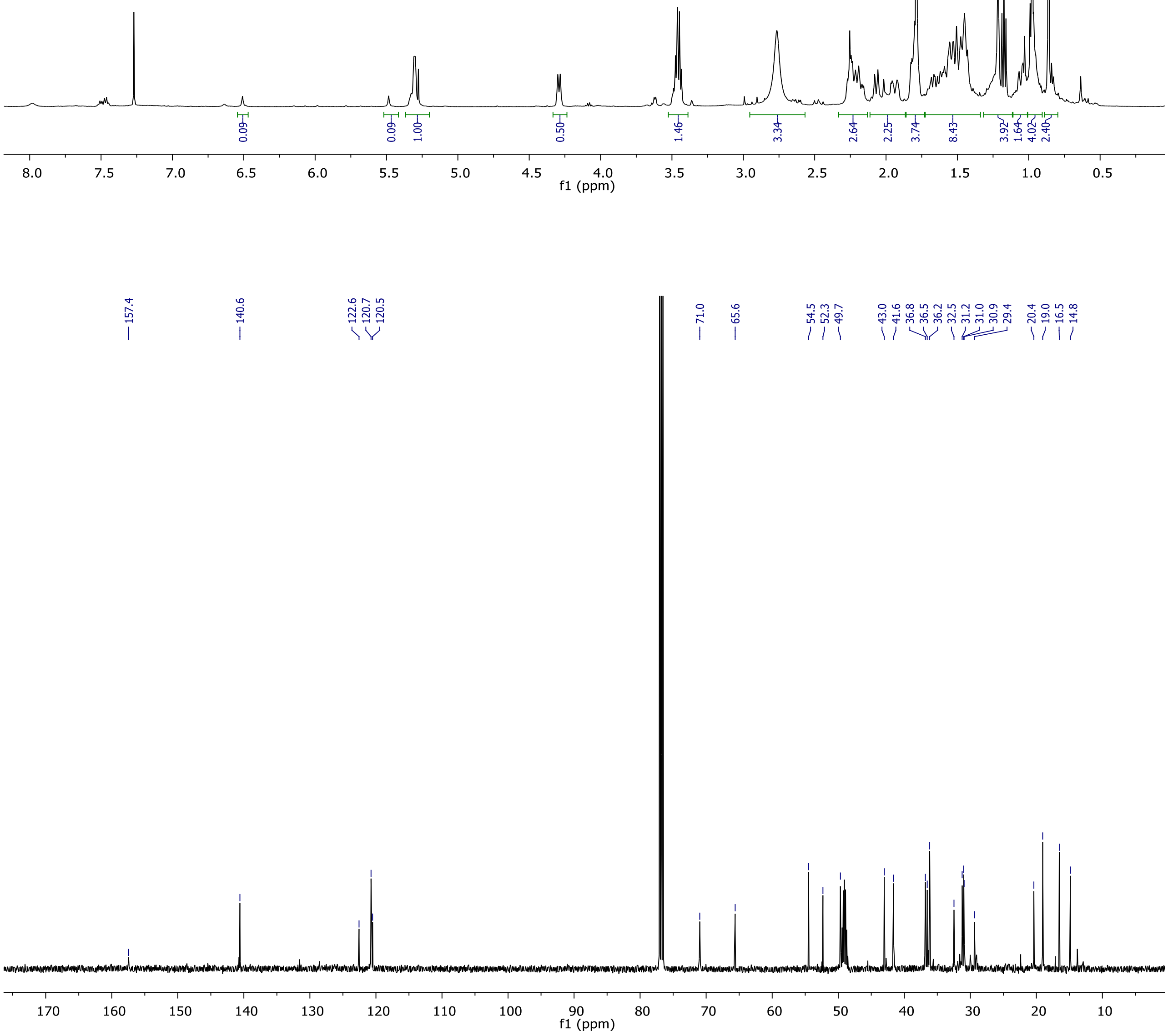

Figure S5. ^{1}H NMR (500 MHz, $CDCl_3$) and ^{13}C NMR (125 MHz, $CDCl_3$) of **7b**.

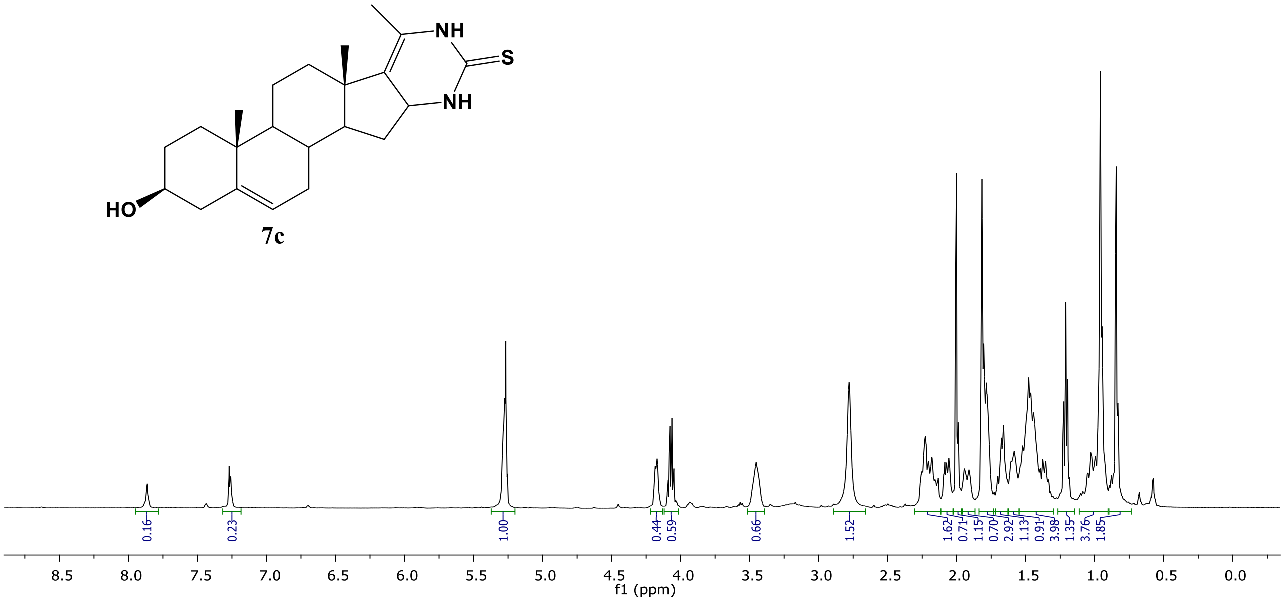

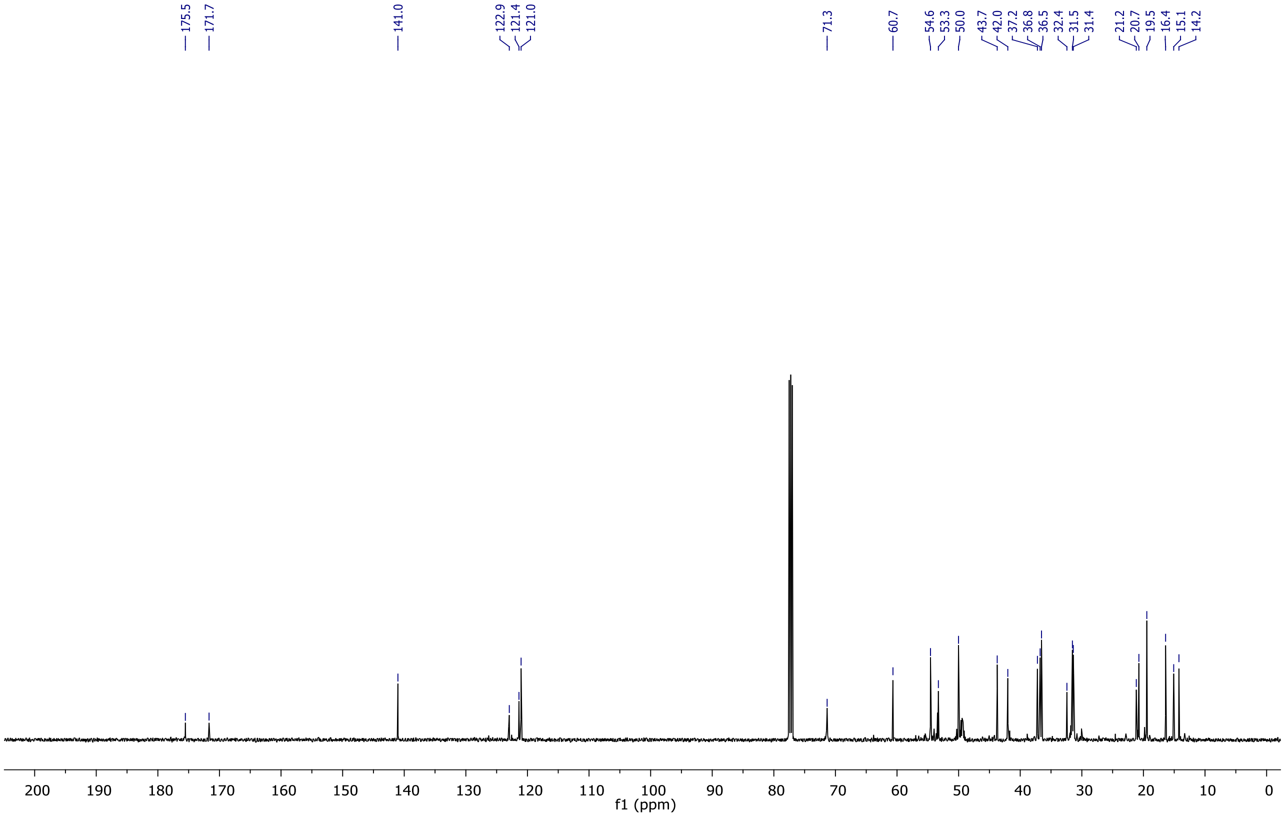

Figure S6. ^{1}H NMR (500 MHz, $CDCl_3$) and ^{13}C NMR (125 MHz, $CDCl_3$) of **7c**.
